# In Silico Hazard Assessment of Ototoxicants Through Machine Learning and Computational Systems Biology

**DOI:** 10.3390/toxics14010082

**Published:** 2026-01-16

**Authors:** Shu Luan, Chao Ji, Gregory M. Zarus, Christopher M. Reh, Patricia Ruiz

**Affiliations:** 1 School of Biological Sciences, Georgia Institute of Technology, Atlanta, GA 30332, USA; 2 Agency for Toxic Substances and Disease Registry, Centers for Disease Control and Prevention, Atlanta, GA 30341, USAgmzarus@gmail.com (G.M.Z.);

**Keywords:** ototoxicity, environmental chemicals, machine learning models, computational toxicology, polychlorinated biphenyls (PCBs), mitotane, molecular target prediction, thyroid-stimulating hormone receptor (TSH), systems biology modeling, virtual screening

## Abstract

Individuals across their lifespan may experience hearing loss from medications or chemicals, prompting concern about ototoxic environmental exposures. This study applies computational modeling as a screening-level hazard identification and chemical prioritization approach and is not intended to constitute a human health risk assessment or to estimate exposure- or dose-dependent ototoxic risk. We evaluated in silico drug-induced ototoxicity models on 80 environmental chemicals, excluding 4 with known ototoxicity, and analyzed 76 chemicals using fingerprinting, similarity assessment, and machine learning classification. We compared predicted environmental ototoxicants with ototoxic drugs, paired select polychlorinated biphenyls with the antineoplastic drug mitotane, and used PCB 177 as a case study to construct an ototoxicity pathway. A systems biology framework predicted and compared molecular targets of mitotane and PCB 177 to generate a network-level mechanism. The consensus model (accuracy 0.95 test; 0.90 validation) identified 18 of 76 chemicals as potential ototoxicants within acceptable confidence ranges. Mitotane and PCB 177 were both predicted to disrupt thyroid-stimulating hormone receptor signaling, suggesting thyroid-mediated pathways may contribute to auditory harm; additional targets included AhR, transthyretin, and PXR. Findings indicate overlapping mechanisms involving metabolic, cellular, and inflammatory processes. This work shows that integrated computational modeling can support virtual screening and prioritization for chemical and drug ototoxicity risk assessment.

## 1. Introduction

Hearing loss is a growing concern affecting people of all ages, and it has become a significant public health issue worldwide [1,2,3,4,5]. According to the World Health Organization, more than 1.5 billion people globally live with some degree of hearing loss, including over 430 million individuals with disabling hearing loss, and this number is projected to increase to nearly 2.5 billion by 2050. The burden of hearing loss is not evenly distributed, with higher prevalence observed among older adults, individuals in low- and middle-income countries, and populations experiencing occupational or environmental exposures.

One often overlooked factor in this public health crisis is exposure to ototoxicants—substances such as pharmaceuticals, household products, and environmental chemicals that can damage our ears and lead to hearing loss [1,6,7,8,9,10,11]. Each year, millions of individuals worldwide are exposed to known or suspected ototoxic agents, including commonly prescribed medications (e.g., chemotherapeutics, antibiotics, diuretics), industrial chemicals, and persistent environmental pollutants. Certain populations—such as cancer patients, industrial workers, military personnel, agricultural workers, and communities residing near contaminated sites—experience disproportionately higher exposure to ototoxic risk factors.

These hazardous substances may directly injure the structures in the ear or disrupt the auditory nerve, which plays a crucial role in our ability to hear. Epidemiological and occupational studies indicate that chemical exposures often occur concurrently with other auditory stressors, such as noise, aging, or pre-existing health conditions. Additionally, several studies have reported that ototoxicants may exacerbate hearing loss when combined with noise exposure, resulting in synergistic effects that substantially increase the risk and severity of auditory impairment, particularly in occupational and environmental settings [11,12].

Despite the growing recognition of these risks, substantial gaps remain in understanding how environmental chemicals contribute to ototoxicity, particularly for non-pharmaceutical substances. Many common substances, from industrial waste to everyday household products, may present concealed dangers to our hearing health. While the Agency for Toxic Substances and Disease Registry (ATSDR) has summarized the ototoxic risks of several substances in its Toxicological Profiles [3], the majority of environmental chemicals lack auditory-specific toxicological data, and many substances lack hearing-specific research, making it challenging for public health professionals and researchers to accurately assess the extent of the issue [13]. This paucity of substance-specific auditory endpoints constrains public health professionals from being able to protect communities from adverse health effects associated with hazardous environmental exposures [13].

Computational toxicology approaches have increasingly been used to address such data gaps; however, existing computational studies of ototoxicity exhibit several important limitations. Many QSAR and machine learning models rely on small, heterogeneous datasets or toxicity endpoints that are not specific to auditory outcomes, often using general neurotoxicity or cytotoxicity as surrogates for hearing loss. In addition, most existing models focus on isolated chemical structure–activity relationships without incorporating biological context, limiting mechanistic interpretability. Biological system-level interactions and pathway-based effects relevant to auditory function are rarely examined, reducing the usefulness of these models for regulatory prioritization and hypothesis-driven research.

ATSDR’s applied toxicology programs are designed to address many data gaps [14,15]. To overcome the limitations of existing computational studies and address the substantial ototoxicity data gap for environmental chemicals, we employed innovative computational toxicology predictive models and computational systems biology tools to focus on environmental chemicals [16,17,18,19,20,21,22,23]. Among these approaches, machine learning models and quantitative structure–activity relationship (QSAR) models play essential roles in predicting the ototoxicity of chemicals, even when data are limited [24,25]. Machine learning models can analyze large datasets to identify structural patterns and relationships between chemical properties and biological outcomes related to hearing loss [24]. By training these models on existing toxicity data, we can make informed predictions about new or untested chemicals based on their structural characteristics [19,20].

Similarly, QSAR models leverage known chemical structures to predict biological activity by establishing correlations between molecular features and their potential effects on living organisms [19,20,23]. In contrast to prior QSAR-only ototoxicity studies, the present work integrates predictive modeling with computational systems biology analyses, enabling evaluation of predicted ototoxicants within the context of auditory-relevant molecular pathways and biological networks. By incorporating genomic-, proteomic-, and pathway-level information, this approach provides mechanistic insight into how environmental chemicals may disrupt interconnected biological processes essential to auditory function or exacerbate existing vulnerabilities. This systems-level integration represents a conceptual and methodological advance beyond traditional structure–activity approaches.

The objectives of this study are to support public health decision-making by systematically identifying and prioritizing environmental chemicals with potential ototoxic risk using an integrated computational toxicology framework. Specifically, we aim to (i) apply machine learning-based QSAR models to screen data-poor environmental chemicals for predicted ototoxicity, (ii) incorporate computational systems biology analyses to place predicted hazards within auditory-relevant molecular pathways and biological networks, and (iii) reduce critical data gaps that currently limit hearing-related risk evaluation for environmental contaminants. This work contributes a novel, biologically informed approach that advances beyond existing QSAR-only or single-endpoint computational studies by linking scalable chemical screening with mechanistic interpretation. 

## 2. Materials and Methods

**Figure 1:**
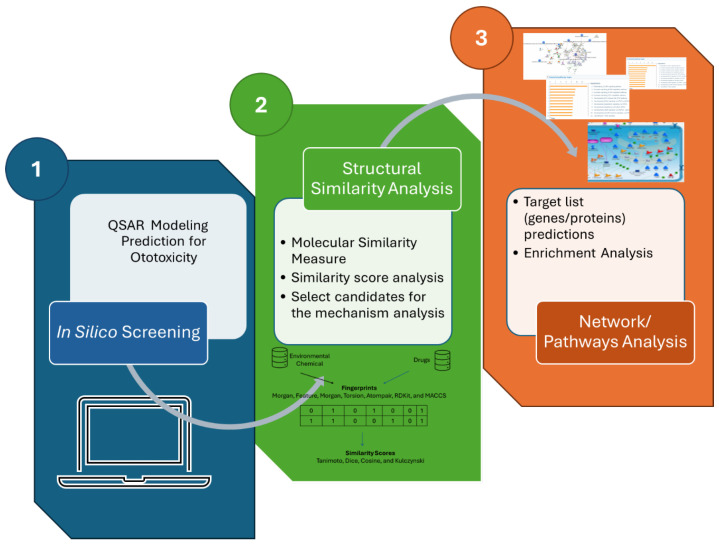
Predictive Workflow for Ototoxicity Assessment Using in Silico Methods. It begins with in silico screening (QSAR Modeling) to apply predictive models based on known ototoxic and non-ototoxic drugs. Next, Structural Similarity Analysis identifies compounds with similarities to established ototoxic drugs through cheminformatics tools. The process concludes with Network and Pathways Analysis, mapping selected compounds to biological targets and conducting enrichment analysis to identify affected pathways and processes related to ototoxicity. This integrated approach facilitates the identification and assessment of potential ototoxic risks associated with environmental chemicals.

In this study, we employed a systematic approach to predict ototoxicity using in silico methods (Figure 1). First, we conducted QSAR modeling to assess the ototoxic potential of a set of environmental chemicals by applying a published predictive ototoxicity model based on a dataset of known ototoxic and non-ototoxic drugs (step 1 in blue color, Figure 1). Following this, we performed a structural similarity analysis to identify compounds with structural similarities to established ototoxic drugs, utilizing cheminformatics tools to calculate similarity scores and generate a similarity matrix (step 2, Figure 1, in green color). Finally, we conducted network and pathway analysis by mapping the selected compounds to biological targets using relevant databases, constructing interaction networks to visualize relationships, and performing enrichment analysis to identify significantly affected pathways and biological processes associated with ototoxicity (step 3 in orange color, Figure 1). This comprehensive methodology allows for a robust risk assessment of potential ototoxic compounds.

### 2.1. QSAR Model Prediction for Ototoxicity

We utilized the QSAR model developed by Huang et al. (2021) [24] to predict ototoxicity in a set of environmental chemicals. This previously published and validated consensus QSAR model was trained using a large, curated dataset of 2807 compounds, including 1102 ototoxicants and 1705 non-ototoxicants, annotated based on experimental in vivo, in vitro, and clinical evidence of hearing-related effects. The modeling dataset was divided into a training set (2121 compounds), an internal test set (236 compounds), and a fully independent external validation set (450 compounds), as reported by Huang et al. (2021) [24].

Model performance was evaluated by Huang et al. (2021) [24] using five-fold cross-validation, an internal test set, and an external validation set. The consensus model demonstrated strong predictive performance, with accuracy = 0.96, sensitivity = 0.93, specificity = 0.98, AUC = 0.98, and MCC = 0.91 on the test set, and accuracy = 0.90, sensitivity = 0.82, specificity = 0.93, AUC = 0.92, and MCC = 0.73 on the external validation set. In the present study, the model was applied without retraining or recalibration to preserve consistency with the original validation and to avoid introducing bias in the absence of sufficient hearing-specific ototoxicity data for environmental chemicals. The drug-induced ototoxicity QSAR model developed by Huang et al. (2021) [24] was applied as a screening-level hazard identification tool to prioritize environmental chemicals based on intrinsic structure–activity relationships associated with ototoxicity. Because the model predicts chemical hazard rather than exposure- or dose-dependent risk, its application in this study is intended for chemical prioritization rather than quantitative risk assessment. Model parameters, including decision thresholds, were retained as originally defined to preserve model integrity and comparability with the original validation, as robust hearing-specific ototoxicity datasets for environmental chemicals are currently insufficient to support exposure-informed recalibration. All evaluated chemicals were assessed within the model’s applicability domain, and predictions were interpreted conservatively as indicators of relative ototoxic potential. This analysis is not intended to constitute a human health risk assessment or to estimate real-world exposure-related risk.

**Figure 2:**
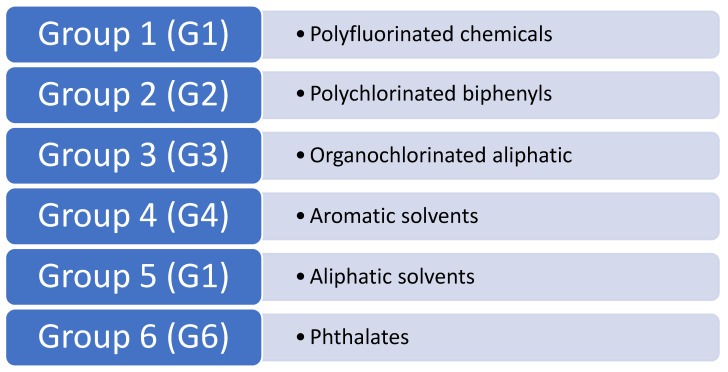
Six chemical groups were identified for QSAR modeling screening for potential ototoxicity. The six chemical groups represent compounds originating from common industrial, commercial, and consumer products—such as firefighting foams, electrical equipment, fuels, solvents, and plasticizers that enter the environment through manufacturing effluents, wastewater discharge, atmospheric emissions, and degradation of consumer materials.

We compiled our chemical dataset by identifying potential environmental ototoxicants from our toxicological profiles and relevant literature [3,7,10,26,27,28,29]. The initial pool of candidate chemicals was generated by prioritizing substances with documented or suspected human exposure, regulatory or public health relevance, and reported associations with neurotoxicity, endocrine disruption, or auditory-related outcomes. We selected chemicals for inclusion based on documented or suspected human exposure, availability of structural information required for computational modeling, and documented or suspected toxicological data from human or animal studies, while excluding chemicals that cannot be structurally modeled (e.g., metals, salts, and chemical mixtures), resulting in a final dataset of 80 environmental chemicals. This selection strategy was designed to reflect real-world exposure scenarios and to focus on chemicals of public health concern for which hearing-related effects remain under-characterized. These chemicals were subsequently categorized into six distinct groups based on their chemical classification. The identified groups are as follows: Group 1 (G1) includes polyfluorinated chemicals; Group 2 (G2) polychlorinated biphenyls; Group 3 (G3) organochlorinated aliphatics; Group 4 (G4) aromatic solvents; Group 5 (G5) aliphatic solvents; and Group 6 (G6) phthalates, as shown in Figure 2. Grouping chemicals by structural class allowed us to evaluate whether specific chemical features or classes were preferentially associated with predicted ototoxic potential. Four chemicals out of the eighty in the dataset with known experimental ototoxicity (toluene, dichloromethane, tetrachloroethylene, trichlorethylene) in the model database were excluded from in silico screening, leaving seventy-six chemicals for QSAR model prediction.

Each compound in our dataset was represented by its Simplified Molecular Input Line Entry System (SMILES) notation. Ototoxicity prediction for the 76 environmental chemicals was conducted using the consensus QSAR model developed by Huang et al. [24] which is accessible on the OCHEM platform at https://ochem.eu//model/profile.do?public_id=890 (accessed 7 January 2026).

The application of the QSAR ototoxicity model involved several steps. First, we formatted the calculated descriptors according to the specifications outlined by Huang et al [24]. Next, we input each compound’s descriptor profile into the model to generate predictions regarding its potential ototoxicity. The results were categorized into “ototoxicity risk” or “not ototoxicity risk” based on established thresholds from Huang et al.’s findings [24]. These thresholds were retained without modification, as the model outputs represent intrinsic chemical hazard rather than exposure- or dose-dependent risk.

Applicability Domain (AD) assessment was performed to ensure prediction reliability and minimize extrapolation beyond the validated chemical space. The AD was defined based on descriptor-space similarity and structural diversity metrics reported by Huang et al. [24], including molecular descriptor ranges and fingerprint-based similarity analysis. Only chemicals falling within the defined AD were retained for interpretation and downstream analyses, while compounds outside the AD were excluded [20,30,31]. The QSAR-predicted environmental ototoxicants were grouped according to their functional groups and structural similarity.

To avoid redundancy, detailed model development procedures were not reproduced in full in the Methods; however, key information on training dataset composition, validation strategy, performance metrics, and applicability domain assessment is summarized in Supplementary Table S1, with full methodological details available in Huang et al. (2021) [24].

### 2.2. Structural Similarity Analysis

#### 2.2.1. Molecular Similarity Measure

To identify structurally similar ototoxic drug molecules from the Huang et al. [24] dataset that correspond to predicted ototoxic environmental chemicals, we used RDKit (version 2022.09.5), an open-source cheminformatics toolkit [32]. Huang et al. [24] compiled a dataset of 1102 ototoxic drugs. For each drug–chemical pair, we computed three types of molecular fingerprints: Morgan, Feature Morgan, and Atompair. Morgan fingerprints capture local atomic neighborhoods; Feature Morgan highlights functionally relevant substructures; and Atompair fingerprints represent global atom-to-atom distance relationships. These fingerprints were chosen for their complementary ability to convey both localized and more global structural pertinent features to molecular similarity. Pairwise structural similarity was quantified using the Tanimoto coefficient for each fingerprint, yielding three similarity scores per drug–chemical pair. To avoid bias from trivial matches and potential data leakage, we excluded predicted-positive environmental chemicals with exact structural matches (based on SMILES) to compounds in the Huang et al. [24] database that have experimental positive ototoxicity before similarity computation (Figure 1, step 2, green color).

#### 2.2.2. Similarity Score Analysis

Downstream data analysis and visualization were performed using the R 4.5.2 software. Heatmap and scatterplot visualizations were generated with the ggplot2 4.0.1 package. We calculated background Tanimoto similarity scores for each fingerprint using all molecules in the Huang et al. [24] database. We subsequently fitted a Gaussian kernel function to each fingerprint similarity score distribution. From this, we derived the empirical cumulative density function (ECDF), which gives the proportion of similarity scores less than or equal to a given value. For each unique similarity score pair, we then calculated the *p*-value as the probability of observing a score higher than the similarity score, defined as: *p* = 1 − ECDF (similarity score). The Tanimoto similarity and statistical significance thresholds were selected based on established practices in similarity-based cheminformatics and read-across. While higher Tanimoto cutoffs (e.g., ≥0.5–0.7) are often used to identify near-identical analogs, moderate thresholds in the range of ~0.3–0.5 are widely applied in screening-level analyses to capture meaningful structural similarity across diverse chemical spaces, particularly when combined with complementary evidence [33,34,35]. Previous work has also demonstrated that the interpretation of Tanimoto similarity scores is context-dependent and should be supported by statistical testing rather than by fixed cutoffs alone [33]. Accordingly, a Tanimoto similarity cutoff of ≥0.4 was selected as a conservative and inclusive criterion for exploratory similarity assessment.

To further reduce the likelihood of spurious matches, statistical significance was incorporated by evaluating similarity scores against background distributions derived from the full drug database. Nonparametric *p*-values were calculated separately for each fingerprint type, and the combined requirement of Tanimoto similarity ≥ 0.4 and *p*-value < 0.05 was applied to ensure that retained similarities were unlikely to occur by chance. We selected the most similar drug-induced ototoxicity molecules for each group based on a Tanimoto score ≥ 0.4 and a *p*-value < 0.05 for the similarity metrics. These thresholds were applied to support screening-level similarity analysis and mechanistic interpretation rather than definitive structural equivalence or quantitative risk assessment. A final list of similar drugs for each group was compiled by combining all significantly similar drugs identified across different metric combinations.

The agreements among similarity scores were visualized using heatmaps to effectively illustrate the relationships between environmental chemicals and their corresponding drug counterparts.

### 2.3. Target Network and Pathway Analysis

MetaDrug™ (Clarivate, New York, NY, USA) employs cheminformatics tools to identify structurally similar compounds in its database and report their interactions with proteins. This enables users to predict biological effects, as illustrated in Figure 1 (step 3, highlighted in orange). Our predicted ototoxicants were queried against a database of approximately 700,000 manually annotated compounds linked to around 4500 protein targets associated with small-molecule xenobiotics. We utilized the Accord Chemistry Cartridge (Accelrys, San Diego, CA, USA) to perform a similarity search using fragment-based fingerprints. Thus, a list of compounds can be generated based on exact matches or high similarity scores (using the Tanimoto coefficient). We conducted a 70% match similarity analysis to focus on literature-reported targets and those predicted by QSAR models for further pathway analysis.

The identified protein targets were then used for enrichment analysis within Canonical Pathway Maps from MetaCore (Clarivate, New York, NY, USA) gene ontologies, allowing us to rank cellular pathways influenced by the uploaded chemicals. The significance of enrichment analysis was determined using *p*-values calculated via the hypergeometric distribution (Fisher’s exact test), resulting in a ranked list of maps that summarize potential toxic effects at the systems-biology level.

We expanded the protein target list for potential ototoxicants by constructing a network based on protein–protein interactions and identifying nearest neighbors. This network draws on curated interactions documented in MetaDrug™, which maps individual chemicals and proteins to functional ontologies such as gene–disease associations and mechanisms of toxicity. Through this process, biological networks are reconstructed, connecting component nodes into meaningful clusters.

## 3. Results

### 3.1. In Silico Screening (QSAR Ototoxicity Modeling)

Utilizing the QSAR model developed by Huang et al. [24] the QSAR modeling approach successfully predicted the ototoxic potential of various chemicals. The QSAR model identified 18 environmental chemicals with potential ototoxic effects among the 76 chemicals screened. Combining the four chemicals with known ototoxicity yielded a list of 22 environmental ototoxicants. These were subsequently categorized into the six distinct groups as follows: Group 1 (G1) includes 11 polyfluorinated chemicals; Group 2 (G2) encompasses 3 polychlorinated biphenyls; Group 3 (G3) consists of 2 organochlorinated aliphatics; Group 4 (G4) comprises 2 aromatic solvents; Group 5 (G5) contains 3 aliphatic solvents (available experimental ototoxicity data); and Group 6 (G6) consists of 1 phthalates, as shown in Figure 2. The four chemicals with known ototoxicity included one in G4 and three in G5.

In addition to shared structural features, each group reflects common product uses and environmental sources. Polyfluorinated chemicals (G1) are associated with firefighting foams, nonstick coatings, and stain-resistant materials and are released via industrial effluents and wastewater. Polychlorinated biphenyls (G2) originate from legacy uses in electrical equipment and building materials and persist in the environment through leaching and sediment resuspension. Organochlorinated aliphatics (G3) and aromatic/aliphatic solvents (G4–G5) are widely used in fuels, paints, degreasers, and cleaning products and are released through industrial discharges, spills, and atmospheric emissions. Phthalates (G6) are used as plasticizers in consumer products and enter the environment through product degradation and wastewater pathways.

The predictive consensus ototoxicity model developed by Huang et al. [24] which used six machine learning models, achieved an overall accuracy of 95% during cross-validation. The external validation set confirmed the model’s robustness, yielding an accuracy of 90%. All the predictions of the environmental chemicals were inside the AD. Within these six groups, the model accurately predicted the known ototoxicants: toluene, dichloromethane, tetrachloroethylene, trichlorethylene (in groups 4 and 5), as well as non-ototoxicant tetrachloromethane, included in our study dataset and reported in the literature.

These results indicate that the developed QSAR model is a reliable tool for predicting ototoxicity.

### 3.2. Similarity Analysis Between Environmental Chemicals and Ototoxic Drugs

**Figure 3:**
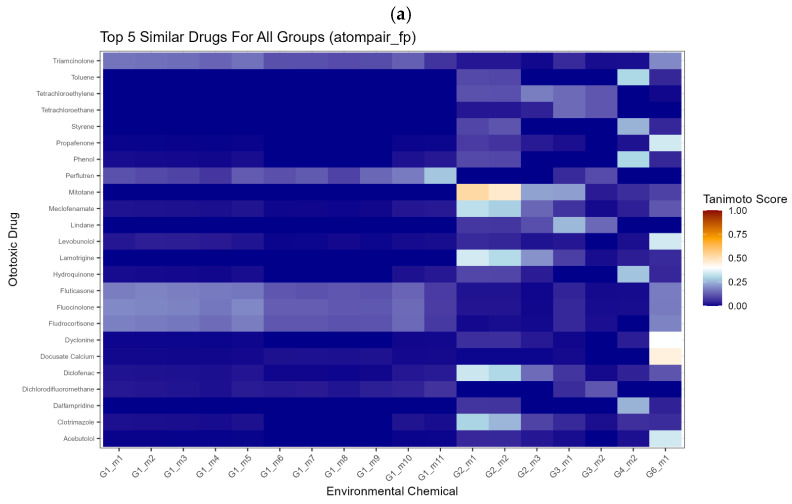

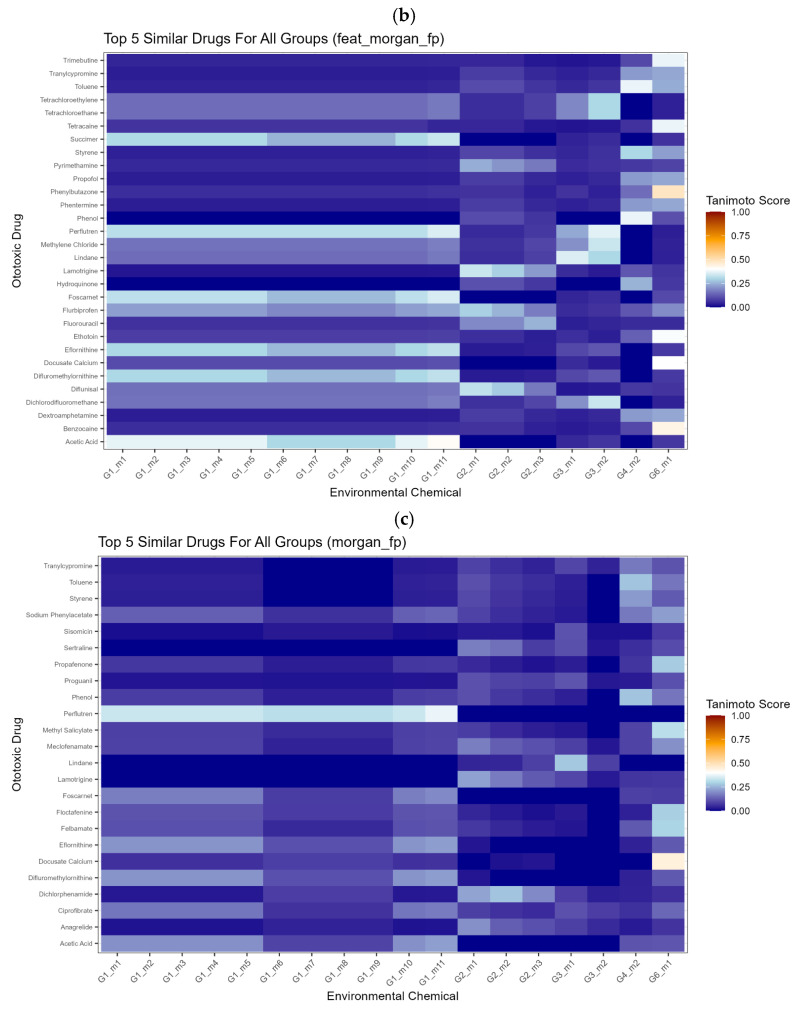
Identification of a significant environmental chemical–ototoxic drug pair based on molecular similarity calculated using (**a**) atom-pair fingerprint; (**b**) feat morgan fingerprint; and (**c**) morgan fingerprint.

For the 18 environmental chemicals with predicted ototoxicity, similarity scores were calculated and heatmaps were generated for visualization. Heatmaps illustrate the top five ototoxic drugs from Huang et al.’s [24] dataset that show the highest similarity scores with the predicted ototoxic environmental chemicals (Figure 3). Group 1 (polyfluorinated chemicals) is shown to have a high consensus similarity score with perflutren across the molecular fingerprints. Group 2 (polychlorinated biphenyls) shows strong overall structural similarity to the antineoplastic agent mitotane. Other drug–chemical pairs within the groups include lindane (Group 3), styrene (Group 4), and phenylbutazone (Group 6).

These findings suggest potential candidates for further investigation regarding their ototoxic potential, based on the known mechanism of action of the drugs’ ototoxicity. Among the six chemical groups, we conducted further analysis on Group 2, specifically polychlorinated biphenyls (PCBs), with a focus on PCB 177 and its similar identified ototoxicant drug, mitotane. This environmental chemical–drug pair met the significance criteria for structural similarity: polychlorinated biphenyl (PCB 177; molecule 1 from group 2) and mitotane. Similarity was determined using the atom-pair fingerprint method, yielding a Tanimoto score of 0.525 (greater than the 0.4 threshold), with strong statistical support (adjusted *p* = 1.29 × 10^−19^).

### 3.3. Target Network and Pathways Analysis

**Figure 4:**
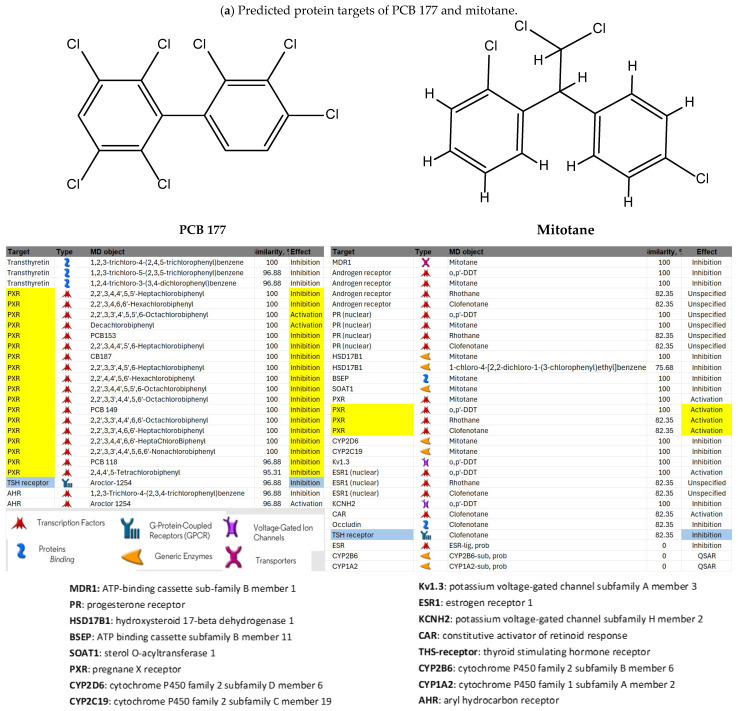

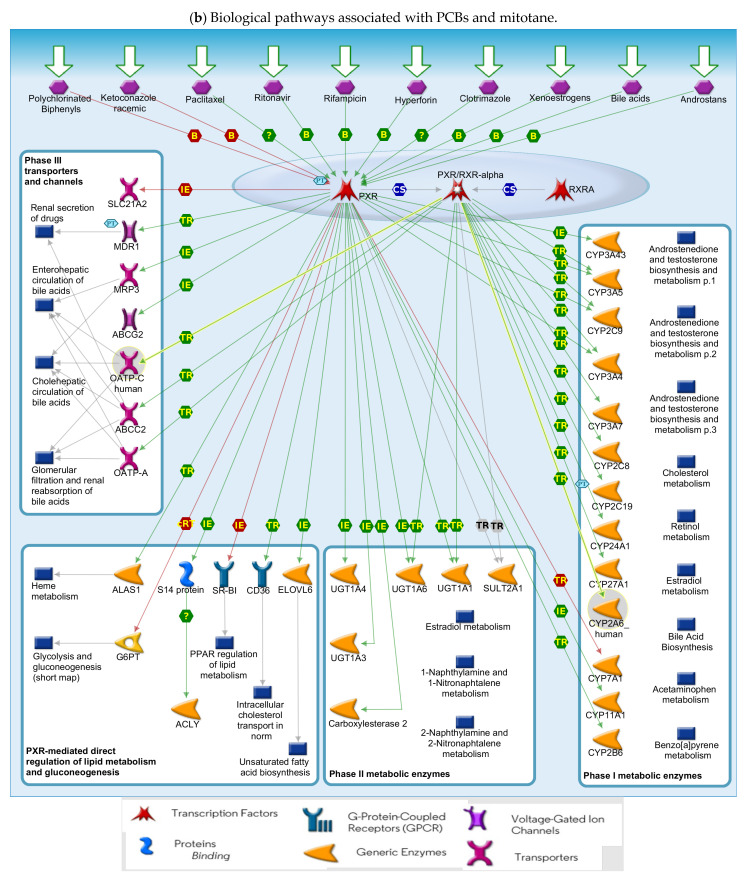
Predicted protein targets and associated biological pathways of PCB 177 and mitotane.

**Figure 5:**
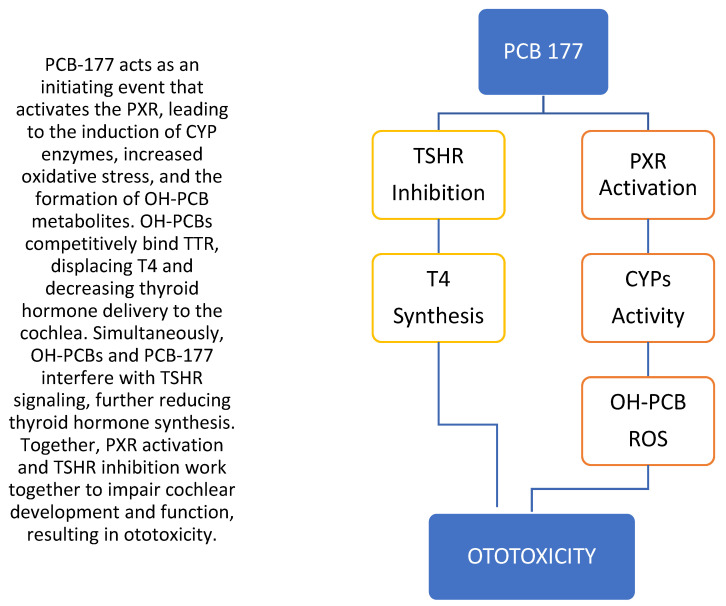
Proposed mechanism of PCB-177-induced ototoxicity via PXR activation and thyroid hormone disruption by mitotane common pathways. PCB-177, 2,2′,3,3′,4′,5,6-heptachlorobiphenyl; PXR, pregnane X receptor; CYP, cytochrome P450; OH-PCB, hydroxylated PCB; TTR, transthyretin; T4, thyroxine; TSHR, thyroid-stimulating hormone receptor.

Using MetaDrug^TM^ software, PCB 177 and mitotane were mapped to their predicted protein targets and subsequently linked to biological pathways (Figure 4). The merged interaction network illustrates computationally inferred relationships between chemical structures, their molecular targets, and downstream pathways, providing a hypothesis-generating mechanistic context for potential points of overlap between environmental chemicals and ototoxic drugs. This analysis suggests that the transthyretin, Pregnane X receptor (PXR), thyroid-stimulating hormone receptor (TSHR), and Aryl hydrocarbon receptor (AhR) are protein targets for PCB 177, and mitotane shares the thyroid-stimulating TSHR and PXR targets. Based on these predicted overlaps, we hypothesize that common signaling pathways may converge through PXR and TSHR, providing a plausible biological link between PCB 177 and known ototoxic drugs. Figure 5 presents a conceptual mechanistic framework derived from shared predicted targets and pathway annotations, illustrating how PCB 177 could plausibly engage biological processes similar to those implicated in mitotane-associated ototoxicity. This framework is intended to generate testable hypotheses rather than to establish causality and reflect integration of database-derived interactions and prior literature, rather than direct experimental evidence.

Overall, network and pathway analyses suggest potential biological processes, including pathways related to metabolism, endocrine signaling, and cellular stress, that may be influenced by PCB 177 and warrant further experimental investigation.

## 4. Discussion

The present study employed a comprehensive in silico screening-level framework to evaluate the ototoxic potential of various environmental chemicals using QSAR modeling, structural similarity analysis, and network and pathway analysis. While QSAR and similarity analyses support the prioritization of chemicals with potential ototoxic hazard, the target network and pathway analyses were conducted to provide a biologically plausible, hypothesis-generating context rather than confirmatory mechanistic evidence.

Pharmaceuticals and environmental chemicals differ markedly in exposure routes, dose ranges, and metabolic contexts, which can influence real-world ototoxic outcomes. While the QSAR model used in this study was originally developed from drug-induced ototoxicity data, its predictions reflect intrinsic chemical hazard rather than exposure-specific risk and they are therefore appropriate for screening-level prioritization. Application of a drug-derived model to environmental chemicals introduces uncertainty related to extrapolation across chemical space; to mitigate this limitation, predictions were restricted to the applicability domain and supplemented with computational systems biology analyses to provide biological plausibility and mechanistic context. We acknowledge that chronic low-dose exposure, bioaccumulation, and environmental metabolism are not captured by the current framework, and future integration of toxicokinetic and exposure data will be necessary to refine thresholds and advance toward quantitative risk assessment. Nonetheless, the present approach provides a transparent and reproducible strategy for prioritizing environmental chemicals for targeted experimental follow-up.

The categorization of the predicted ototoxicant chemicals into six structure-based groups (e.g., polyfluorinated chemicals, polychlorinated biphenyls, aromatic solvents, etc.) provides a framework for understanding the structural characteristics associated with ototoxicity. This structural grouping is supported by prior toxicological studies showing that specific chemical classes are more frequently associated with auditory dysfunction [5,6,7]. Many environmental chemicals prioritized in this study originate from widely used industrial, commercial, and consumer products, highlighting ototoxic risk beyond occupational or clinical settings. Polychlorinated biphenyls (PCBs), for example, were historically used in electrical equipment, hydraulic fluids, and building materials; despite bans, they persist in aging infrastructure and contaminated sediments and continue to be released through leaching, volatilization, and sediment resuspension. Other chemical groups examined, including organochlorinated solvents, aromatic and aliphatic solvents, phthalates, and polyfluorinated chemicals, are associated with degreasing agents, fuels, paints, plastics, medical devices, firefighting foams, and water-repellent consumer products. Environmental releases occur through industrial effluents, wastewater treatment discharges, landfill leachate, atmospheric emissions, and urban or agricultural runoff, leading to contamination of air, water, soil, and food webs. These pathways contribute to chronic low-level human exposure and ecosystem disruption through endocrine interference, oxidative stress, and neurodevelopmental effects. Framing ototoxicity within these contamination pathways is essential for upstream risk identification, enabling the prioritization of chemicals, exposed populations, and intervention points to support public health protection and prevent environmentally mediated hearing loss.

The structural similarity analysis suggests important correlations between the predicted ototoxicants by the QSAR model and known ototoxic drugs, particularly within the PCBs group. The focus on PCB 177 and its structural similarity to mitotane is particularly important, as these similarities support prioritization for mechanistic exploration but, by themselves, do not establish shared mechanisms of action.

PCBs are a group of 209 persistent environmental contaminants that share structural similarities but exhibit slight differences in their chemical properties. They are known to induce various health effects and have often been tested for toxicity as complex commercial PCB mixtures (Aroclors); however, environmental exposure typically occurs through a limited number of specific congeners [29]. Recently, the CDC National Report on Human Exposures to Environmental Chemicals, which assesses exposure data from the National Health and Nutrition Examination Survey (NHANES), identified 35 individual PCB congeners in the U.S. population. The ATSDR ranks PCBs among the top hazardous substances on its Substance Priority List (SPL) (SPL 2022), consistently ranking them as one of the highest-priority contaminants. The ATSDR’s SPL is a critical tool for prioritizing research, monitoring, and remediation efforts aimed at reducing risks associated with these harmful substances. This #5 ranking reflects their toxicity, widespread environmental contamination, and potential for human exposure through various pathways, including dietary sources and occupational settings. PCBs are also frequently found alongside other neurotoxic substances on the SPL. These neurotoxicants could also be ototoxic substances.

PCB 177 is a non-dioxin-like PCB (NDL-PCB), characterized by the absence of a planar “dioxin-like” structure due to ≥2 ortho chlorine substitutions (2,2′,6,6′), which twist the biphenyl rings. NDL-PCBs constitute the majority of PCBs in the environment and human tissues, representing ~80–85% of the 209 congeners. Because of their large number, researchers and regulatory agencies rely on a reference set of indicator NDL-PCBs (28, 52, 101, 138, 153, and 180) to represent long-term human body burdens, rather than monitoring all 197 congeners individually. However, significant data gaps remain regarding the ototoxic potential of PCBs, as few congener-specific studies have evaluated their effects on hearing outcomes. Moreover, most research has examined PCB mixtures or commercial formulations (Aroclors), making it difficult to isolate the ototoxic effects of individual congeners such as PCB 177. Thus, the predicted ototoxicity of PCB 177 was examined in conjunction with mitotane, which was among the top ototoxic drugs with the closest structural similarity to Group 2. Mitotane (o,p′-DDD) is a well-established antineoplastic agent known to cause ototoxicity. It is an isomer of the insecticide DDD and a chemical congener of DDT [36,37,38]. Due to its direct cytotoxic effects on the fascicular and reticular zones of the adrenal cortex, this drug is used in the treatment of unresectable adrenal cortical carcinoma (ACC) [38,39,40].

Mitotane and PCBs share structural similarities characterized by chlorine atoms attached to carbon backbones [41]. These structural features contribute to their lipophilicity, facilitating their accumulation in biological tissues. They also facilitate their interaction with various biological receptors, leading to significant physiological effects [7,10]. Our findings are consistent with the existing literature, which has documented the ototoxic effects of PCBs and organochlorinated pesticides’ structural analogs [5,6]. Mitotane and PCBs are endocrine disruptors associated with numerous chronic health conditions [36,42]. They interfere with hormonal systems through various mechanisms. Specifically, mitotane, which is employed in the treatment of ACC, disrupts adrenal function and can result in thyroid dysfunction, hypogonadism, and various metabolic disorders [36,38]. In contrast, PCB 177, a persistent organic pollutant, impacts endocrine systems by mimicking or interfering with hormones, leading to reproductive, neurological, and developmental disorders [26,42]. Structural similarity alone does not imply shared toxicity pathways and must be interpreted cautiously.

Our network and pathway analyses were conducted to explore potential literature-supported biological pathways linking PCB 177 and mitotane. The identification of overlapping predicted network targets, including TSHR, PXR, TTR, and AhR (Figure 4), is derived from curated databases and literature integration and should be interpreted as hypothesis-generating. The TSHR is critical for regulating thyroid hormone production, which plays a vital role in numerous physiological processes, including auditory function. The two main thyroid hormones, thyroxine (T4) and triiodothyronine (T3), are synthesized using iodine and amino acids. T3 is the more active hormone, while T4 serves primarily as a precursor that is converted into T3 in tissues [43,44].

Studies indicate that mitotane can inhibit thyroid hormone synthesis by disrupting the signaling pathways associated with the TSHR [45,46]. Inhibition of the TSHR can reduce thyroid gland stimulation, leading to lower levels of thyroid hormones (T3 and T4) [41,47,48,49,50,51,52,53]. A deficiency in thyroid hormones or iodine has been linked to hearing impairments, which result from structural and functional alterations in the inner ear [48,51,53,54]. Some individuals with hypothyroidism have reported experiencing tinnitus. The relationship between thyroid hormone levels and tinnitus is complex and may involve changes in inner ear fluid dynamics or neural excitability due to insufficient thyroid hormone action. A deficiency of T4 or T3 can lead to cochlear dysfunction, which affects hair cells and auditory pathways. Inhibition of the TSHR and subsequent low thyroid hormone levels during critical developmental periods may lead to structural abnormalities in the cochlea, potentially resulting in hearing impairments. Similarly, studies have demonstrated that PCBs can interfere with TSHR signaling by mimicking or blocking endogenous thyroid hormones [43,52,55,56,57]. For instance, PCB exposure has been linked to altered T4 levels in serum, suggesting interference with normal endocrine function. Such disruptions can lead to developmental issues in auditory structures during critical growth periods [26,42,47,55,56,58]. Individuals with low thyroid hormone levels may have an increased risk of ototoxicity, particularly when exposed to a combination of certain drugs or other environmental ototoxicants and noise.

As illustrated in Figure 4, our analysis also suggests that the Pregnane X Receptor (PXR) is a potential molecular target for PCBs and mitotane (Figure 4). PXR plays a crucial role in regulating detoxification processes. PXR controls the production of proteins involved in all three stages of the detoxification mechanism, including the CYP450 enzymes (i.e., CYP3A4), phase II enzymes (i.e., glutathione S-transferase), and phase III transport uptake and efflux proteins, like OATP2 and MDR1, and drug–drug interactions [59,60]. PXR is also involved in various biological processes, including lipid metabolism and thyroid hormone homeostasis. It binds to hormone response elements on DNA, thereby eliciting the expression of gene products [27,59,60,61].

By modulating PXR activity, mitotane and PCB 177 may alter the expression levels of genes responsible for drug metabolism and detoxification pathways involving CYP450 enzymes [27,36,59,60].

The activation of PXR can have profound adverse effects on auditory systems, particularly in the cochlea, which may lead to hearing loss. While PXR activation is generally associated with protective roles in detoxification and cellular stress responses, dysregulation or inappropriate activation can result in significant harm. A major concern is the accumulation of toxic metabolites resulting from the altered expression of cytochrome P450 enzymes and transport proteins. This accumulation can directly damage cochlear cells, especially hair cells, which are essential for sound transduction. Additionally, although PXR activation can enhance the expression of antioxidant enzymes, excessive activation may disrupt the balance of oxidative stress responses, leading to increased levels of reactive oxygen species (ROS). This oxidative stress can overwhelm cellular defenses, leading to oxidative damage in cochlear cells and contributing to hair cell death. Furthermore, chronic inflammation driven by inappropriate PXR activation can elevate pro-inflammatory cytokines, leading to cellular dysfunction and progressive hearing impairment.

Moreover, PXR activation influences apoptotic pathways and dysregulation, which can lead to an imbalance in cell survival and death signals, resulting in premature cochlear cell death. It could also lead to the survival and propagation of damaged cells. This loss of critical cell types impairs auditory function. The inner ear’s reliance on precise ion homeostasis means that changes in PXR activity can disrupt the expression and function of ion channels, compromising the electrochemical gradients necessary for sound transduction. Additionally, during critical developmental periods, proper PXR activation is essential for the differentiation and maturation of sensory cells; any dysregulation during these windows can lead to structural abnormalities that manifest as hearing loss later in life. Lastly, certain drugs and xenobiotics, such as PCBs, that activate or inhibit PXR may pose a risk of ototoxicity, particularly at high doses or prolonged exposure, further complicating the relationship between PXR activation and cochlear health. Collectively, these factors suggest the complex role of PXR in auditory health and the potential consequences of its dysregulation on hearing loss. PXR’s direct role in ototoxicity remains incompletely characterized. The proposed pathways described here, therefore, represent plausible but unconfirmed links.

Furthermore, our analysis suggested additional targets interacting with PCB 177, including the TTR and AhR. The activation of AhR can result in the induction of cytochrome P450 enzymes involved in chemical metabolism; however, it may also lead to an increased production of reactive oxygen species (ROS), similar to PXR, which can contribute to cellular damage within the auditory system. PCBs are well-documented AhR agonists that can disrupt normal cellular signaling pathways by binding to this receptor with high affinity. The activation of AhR by PCBs has been linked to oxidative stress responses that could exacerbate ototoxic effects through mechanisms such as inflammation or apoptosis within cochlear cells. These processes are biologically relevant to auditory injury but have not been directly demonstrated for PCB 177-specific ototoxicity.

TTR plays a critical role in auditory function through various mechanisms that underscore its physiological and pathological significance [62,63,64]. As a primary transporter of thyroid hormones such as T4, TTR influences the availability of thyroid hormones, which are essential for developing and maintaining the auditory system, including the cochlea and auditory pathways [64]. Disruptions in TTR’s transport function may lead to altered thyroid hormone levels, which can potentially impact auditory health. Furthermore, TTR is involved in cochlear health, particularly in relation to hereditary transthyretin-related amyloidosis (ATTR) [65,66]. This condition, caused by mutations in the TTR gene, accumulates amyloid fibrils in various tissues, including the cochlea [46,47]. Such accumulation can impair cochlear function and contribute to sensorineural hearing loss, affecting auditory signal processing. Research indicates that cochlear implants can be an effective rehabilitation strategy for individuals with severe hearing loss due to ATTR-related cochlear damage. Emerging studies also suggest that TTR has neuroprotective effects, potentially supporting the health of the auditory nerve and neuronal function. Together, these mechanisms highlight TTR’s crucial role in maintaining auditory health and its impact on hearing impairments under pathological conditions [67,68]. The relevance of TTR-mediated mechanisms to PCB-induced auditory toxicity remains a hypothesis requiring experimental testing.

PCBs are endocrine disruptors, particularly affecting TH signaling. TTR is crucial for transporting T4 to the brain and plays a significant role in regulating free T4 levels in the extracellular space [69,70]. Disruption of TTR function due to PCB exposure may hinder T4 delivery across the blood–brain barrier, potentially leading to altered TH levels in the brain [71]. In utero exposure to polychlorinated biphenyls (PCBs) has been linked to long-term auditory system vulnerabilities. In rodent models, prenatal PCB exposure not only impairs recovery from noise-induced hearing loss but also disrupts auditory midbrain organization [56] and causes enduring dysregulation of synaptic transmission in the auditory cortex—effects that occur independently of peripheral hearing thresholds [72]. Moreover, developmental PCB exposure has been shown to induce permanent low-frequency hearing deficits that persist into adulthood [73]. Specifically, prenatal PCB exposure in mice impaired recovery from noise-induced hearing loss and altered synaptic organization in central auditory structures, such as the inferior colliculus, suggesting that PCBs sensitize the auditory system to later noise trauma [56]. Thyroid hormones (THs) are essential for normal development of auditory pathways; disruptions in TH transport or availability due to PCB exposure can therefore lead to hearing impairments [56]. The conversion of T4 to the more active T3 is vital for neural development, a process primarily mediated by type II deiodinase (D2). D2 is localized in glial and peri-sensory cells in the brain and cochlea, and it amplifies local T3 levels necessary for cochlear maturation [74]. Genetic deletion of D2 in mice results in profound hearing deficits and cochlear malformation, despite normal systemic thyroid hormone levels, highlighting the crucial role of local T4-to-T3 conversion in auditory development [71].

**Figure 6:**
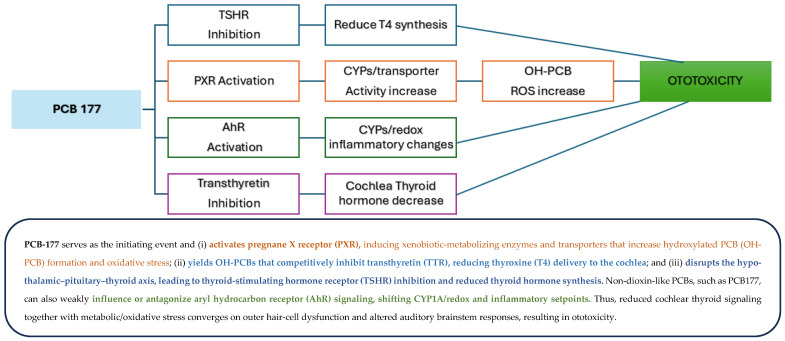
A predicted multi-pathway mechanism by which non-dioxin-like PCB 177 contributes to ototoxicity. NDL-PCBs, non-dioxin-like polychlorinated biphenyls; PCB-177, 2,2′,3,3′,4′,5,6-heptachlorobiphenyl; OH-PCBs, hydroxylated PCBs; PXR, pregnane X receptor; RXR, retinoid X receptor; AhR, aryl hydrocarbon receptor; TTR, transthyretin; TSHR, thyroid-stimulating hormone receptor; T4/T3, thyroxine/triiodothyronine; CYP, cytochrome P450; ABR, auditory brainstem response; ROS, reactive oxygen species.

PCB-induced disruption of thyroid hormone transport or metabolism could therefore increase susceptibility to hearing deficits, particularly following developmental exposure. However, the multi-pathway framework presented in Figure 6 should be interpreted as a conceptual synthesis rather than proof of causality.

Overall, this study distinguishes between well-supported endocrine and auditory biology and computationally inferred target network and pathway relationships, providing a concise mechanistic synthesis that prioritizes PCB-177, other PCBs, and other similar chemicals and pathways for future investigation.

Using QSAR models and computational systems biology tools, we explored potential toxic effects and identified key mechanisms of ototoxicity associated with PCBs and mitotane. Our findings suggest the complex roles of proteins and receptors, including TTR, PXR, TSH, and AhR, in hearing health and ototoxicity. Although this systems biology-generated global network cannot be considered as a proof of causal linkages without further experimental validation, it provides justification for the mechanistic hypothesis. It contributes to a new interpretation linking available published toxicology and disease information domains.

The results of this study highlight the potential for using in silico methods to identify and prioritize environmental chemicals for further ototoxicity evaluation. The predicted ototoxicant chemicals, particularly those within Group 2 (PCBs) and/or their chemical mixtures, warrant further investigation to elucidate their mechanisms of action and assess their risk to auditory health.

### Limitations and Future Directions

While this study provides integrative insights through the combined application of QSAR modeling, structural similarity analysis, and computational system biology tools, several limitations should be considered when interpreting the findings. First, the performance of computational models depends on the quality, scope, and representativeness of the data used for model development. As such, gaps, incompleteness, or biases in available datasets on ototoxicity and chemical–biological interactions may influence prediction reliability and generalizability, particularly for data-poor environmental chemicals.

Second, although computational systems biology approaches enable the integration of large and diverse data sources, they necessarily simplify complex biological processes. Target networks and pathway-based analyses rely on curated databases and literature-derived associations and therefore reflect predicted or inferred relationships rather than experimentally confirmed interactions. Consequently, the mechanistic frameworks proposed in this study should be interpreted as hypothesis-generating models that provide biological plausibility and prioritization context, rather than as evidence of causal mechanisms.

In addition, biological responses to chemical exposures are dynamic and context dependent. Factors such as life stage, sex, genetic susceptibility, pre-existing health conditions, exposure duration, dose, and combined or sequential exposures are not explicitly captured in the present modeling framework. These factors may substantially influence ototoxic outcomes in real-world settings and limit direct extrapolation of computational predictions to population-level risk.

Despite these limitations, the strength of QSAR and computational systems biology tools lies in their ability to efficiently screen large numbers of chemicals, identify potential hazards, and generate testable hypotheses to guide experimental research. Accordingly, experimental validation remains a critical next step. Future studies should include targeted in vitro and in vivo experiments to evaluate the effects of prioritized environmental ototoxicants, both individually and in relevant mixtures, on auditory-related endpoints.

Further research could also leverage transcriptomic, proteomic, metabolomic, and epigenomic approaches to interrogate the specific receptors, pathways, and biological processes identified in this study. Integration of exposure-informed toxicokinetic data and longitudinal study designs would further enhance mechanistic understanding and improve translation of computational modeling findings into risk-relevant insights. Collectively, these efforts will support refinement of predictive models and advance strategies for identifying, preventing, and mitigating environmentally mediated hearing loss.

## 5. Conclusions

Integrating computational toxicology approaches offers an effective strategy for addressing gaps in ototoxicity data, prioritizing chemicals of concern, and generating biologically plausible hypotheses for further investigation. In this study, we combined QSAR modeling, structural similarity analysis, and computational network and pathway approaches to screen environmentally relevant chemicals for ototoxic potential and to explore candidate biological processes associated with auditory system vulnerability.

Our findings suggest that this integrated in silico framework can identify environmental chemicals with predicted ototoxic hazard and support the plausibility of molecular targets and pathways relevant to hearing health. In particular, the analyses are consistent with potential involvement of endocrine regulation and xenobiotic metabolism pathways, including thyroid hormone signaling and nuclear receptor-mediated responses, in the context of PCB exposure and structurally related ototoxic pharmaceuticals such as mitotane. These insights are derived from database-based target predictions, curated pathway annotations, and literature integration, and therefore represent hypothesis-generating interpretations rather than confirmatory evidence of causality.

By prioritizing environmentally relevant, data-poor chemicals and linking them to biologically plausible pathways, this study provides a focused foundation for targeted experimental validation. Future in vitro, in vivo, and omics-based studies will be essential for evaluating the proposed pathways and assessing their relevance to real-world auditory outcomes. Overall, this study demonstrates how computational screening and systems-level analyses can support upstream risk identification, inform public health and regulatory prioritization, and contribute to strategies to prevent environmentally mediated hearing loss and protect population-level auditory health.

## Data Availability

The original contributions presented in this study are included in the article/Supplementary Materials. Further inquiries can be directed to the corresponding author.

## Supplementary Materials

The following supporting information can be downloaded at: https://www.mdpi.com/article/10.3390/toxics14010082/s1, Table S1. QSAR Model Characteristics, Validation, and Applicability Domain.
