# Supplementary material for: In Silico Hazard Assessment of Ototoxicants Through Machine Learning and Computational Systems Biology
Source: Toxics. 2026 Jan 16;14(1):82. doi: 10.3390/toxics14010082 (PMC12845763; doi:10.3390/toxics14010082)
Supplement: Supplementary file 1 [file toxics-14-00082-s001.zip › toxics-4079125-supplementary.pdf]

Supplementary Materials

In Silico Hazard Assessment of Ototoxicants Through Machine Learning and Computational Systems Biology

Summary of the ototoxicity QSAR model developed by Huang et al. (2021), including training dataset composition (chemical sources and endpoint annotation), validation strategy and performance metrics (accuracy, sensitivity, specificity, and AUC), molecular descriptors used for model development, and applicability domain (AD) definition criteria. Table S1 also documents how AD filtering was applied in the present study to restrict predictions to chemicals within the model's validated chemical space.

Table S1. QSAR Model Characteristics, Validation, and Applicability Domain

Category	Description
**Model Reference**	Huang et al. (2021), *In silico prediction of drug-induced ototoxicity using machine learning and deep learning methods*, *Chemical Biology & Drug Design*, DOI:10.1111/cbdd.13894
**Model Type**	Consensus QSAR classification model combining multiple machine learning (ML) algorithms
**Model Purpose**	Screening-level prediction of intrinsic ototoxic hazard based on chemical structure
**Total Dataset Size**	2,807 compounds
**Training Dataset Composition**	2,121 compounds (888 ototoxicants, 1,233 non-ototoxicants; 90% of modeling dataset)
**Internal Test Set**	236 compounds (99 ototoxicants, 137 non-ototoxicants; 10% of modeling dataset)
**External Validation Set**	450 compounds (115 ototoxicants, 335 non-ototoxicants), fully independent of model training
**Endpoint Definition**	Binary classification: ototoxic vs. non-ototoxic, based on experimental in vivo, in vitro, and clinical evidence
**Chemical Diversity Assessment**	Evaluated using molecular weight and ALogP distributions; training, test, and validation sets occupied similar chemical space
**Structural Diversity Metric**	Average Tanimoto similarity index (FCFP4) = 0.135, indicating high structural diversity
**Molecular Representation**	Multiple descriptors sets and fingerprints, including ECFP4, FCFP4, Dragon, CDK, PyDescriptor, and ALogP & OEstate descriptors
**Modeling Algorithms**	Random Forest (RF), Support Vector Machine (LibSVM), and Deep Learning (TCNN, TEXTCNN)
**Internal Validation Strategy**	5-fold cross-validation and an independent internal test set

Category	Description
**Top-Performing Model Selection Criteria**	MCC > 0.30 (5-fold cross-validation) and MCC > 0.75 (test set)
**Consensus Model Construction**	Simple average of six top-performing ML models
**Consensus Model Performance (Test Set)**	Accuracy = 0.96; Sensitivity = 0.93; Specificity = 0.98; AUC = 0.98; MCC = 0.91
**Consensus Model Performance (External Validation)**	Accuracy = 0.90; Sensitivity = 0.82; Specificity = 0.93; AUC = 0.92; MCC = 0.73
**Decision Threshold**	Probability-based classification thresholds as defined in Huang et al. (2021)
**Model Retraining in Present Study**	None; original validated model applied without modification
**Applicability Domain (AD) Definition**	Descriptor-space-based–based applicability domain derived from the training dataset chemical space
**AD Evaluation Methods**	Chemical similarity analysis using FCFP4 fingerprints and descriptor range consistency
**AD Application in Present Study**	Only environmental chemicals falling within the model's applicability domain were retained for interpretation
**Treatment of Chemicals Outside AD**	Excluded from prediction interpretation to minimize extrapolation uncertainty
**Public Availability of Model**	Freely accessible via the OCHEM platform (https://ochem.eu/model/46566321)
**Intended Regulatory Interpretation**	Predictions represent intrinsic ototoxic hazard signals and are not intended to constitute a human health risk assessment
